# Does the pH of the grape seed extract interfere in the biomodification capacity of dentin collagen?

**DOI:** 10.1590/0103-6440202406048

**Published:** 2024-12-16

**Authors:** Marcelo Victor Sidou Lemos, Gabriela Araújo Lourenço, Samuel Chillavert Dias Pascoal, Talita Arrais Daniel Mendes, Sérgio Lima Santiago

**Affiliations:** 1 Graduate Program in Dentistry, Federal University of Ceará, Fortaleza, Ceará, Brazil; 2 University of Fortaleza, Department of Dentistry, Fortaleza, Ceará, Brazil

**Keywords:** collagen, proanthocyanidins, grape seed extract, dentin, pH

## Abstract

The aim of this study was to verify the biomodifying action of 6.5% grape seed extract solutions, with different pH, when applied on dentin collagen. Dentin bars (1.7 mm x 6.0 mm x 0.5 mm) were demineralized for 5 hours in 10% phosphoric acid, and distributed into the following groups: acid solution (pH=4.42); neutral (pH=6.96); alkaline (pH=11.92) and distilled water (pH=6.75). Three-point flexural test (n=10) and mass variation (n=10) were assessed at different periods (baseline, after biomodification, 7 and 14 days of remineralizing solution storage). For qualitative analysis, similar dentin bars were prepared and analyzed using Fourier transform infrared spectroscopy (FT-IR) and Raman Spectroscopy (FT-Raman) before and after 1 hour of immersion in biomodifying solution with different pH. Data were subjected to Shapiro-Wilk normality tests, followed by two-way ANOVA for repeated measures and Tukey's post-test (p<0.05). Alkaline solution was effective in increasing the modulus of elasticity, showing a decrease after 7 days and subsequent stabilization after 14 days of storage. Acid solution group showed a greater increase in the modulus of elasticity immediately after biomodification, but it was not stable over storage. Regarding mass variation, only the acid solution showed an increase in mass after biomodification and 14 days of storage. In relation to FT-IR and FT-Raman, all solutions showed interaction with collagen at some level. Therefore, pH of the solution directly influences the action of the grape seed extract, with satisfactory results being found in both acidic and alkaline solutions.

## Resumo

O objetivo deste estudo foi verificar a ação biomodificadora de soluções de extrato de semente de uva a 6,5%, com diferentes pHs, quando aplicadas no colágeno dentinário. Barras de dentina (1,7 mm x 6 mm x0,5 mm) foram desmineralizadas por 5 horas em ácido fosfórico a 10% e distribuídas nos seguintes grupos: solução ácida (pH=4,42); neutra (pH=6,96); alcalina (pH=11,92) e água destilada (pH=6,75). O teste de flexão de três pontos (n=10) e a variação de massa (n=10) foram avaliados em diferentes períodos (*baseline*, após biomodificação, 7 e 14 dias de armazenamento em solução remineralizante). Para a análise qualitativa, barras de dentina similares foram preparadas e analisadas, antes e após 1 hora de imersão nas soluções de biomodificação, através dos testes de Espectroscopia de infravermelho por transformada de Fourier (FT-IR) e espectroscopia Raman (FT-Raman) . Os dados foram submetidos ao teste de normalidade de Shapiro-Wilk, seguido por ANOVA para medidas repetidas e pós-teste de Tukey (p<0,05). A solução alcalina foi eficaz em aumentar o módulo de elasticidade, mostrando uma diminuição após 7 dias e subsequente estabilização após 14 dias de armazenamento. O grupo da solução ácida mostrou um aumento maior no módulo de elasticidade imediatamente após a biomodificação, mas não foi estável ao longo do armazenamento. Em relação à variação de massa, apenas a solução ácida mostrou um aumento de massa após a biomodificação e 14 dias de armazenamento. Em relação ao FT-IR e FT-Raman, todas as soluções mostraram interação com o colágeno em algum nível. Portanto, o pH da solução influencia diretamente a ação do extrato de semente de uva, com resultados satisfatórios sendo encontrados tanto em soluções ácidas quanto alcalinas.

## Introduction

The increasing demand for aesthetic rehabilitations has made direct restorative procedures using resin composites a routine in the dental practice. However, the adhesive procedure emerges as a critical step, sensitive to the substrate upon which it is applied. Success often hinges on the skill of the operator and the complexity of the procedural steps ^1^. It is widely recognized that the adhesive technique exhibits lesser sensitivity as well as greater efficacy when applied to enamel, owing to its homogeneity. Conversely, dentin presents a more heterogeneous composition, comprising approximately 50% inorganic matter, 30% organic matter, and 20% water content ^1^.

Among the elements comprising the organic matrix, type I collagen stands out, characterized by its triple helix layout and abundance in structural amino acids such as proline ^2^ ^,^ ^3^. While being a sound substrate, dentin is capable of undergoing mineralization. However, during etch-and-rinse adhesive procedures, phosphoric acid can demineralize the superficial dentin (5-8µm), facilitating the subsequent infiltration of adhesive resin monomers into the collagen network. This micromechanical interlocking leads to the formation of a region known as the hybrid layer. Nevertheless, due to the hydrophobic nature of the resin monomers and the moisture within the dentin, complete encapsulation of the fibrils does not occur, leading to the exposure of collagen, rendering it susceptible to the action of collagenolytic proteases like matrix metalloproteinases and cysteine-cathepsins ^2^.

Due to the importance of dentin collagen in the adhesive process, numerous strategies have been developed to enhance its mechanical properties and prolong the lifespan of resin composite restorations. The application of cross-linking reagents is extensively documented in the literature. Such compounds may improve chemical bonding between collagen molecules within its structure, resulting in a stiffer collagen matrix that is degradation-resistant over time. Regardless of its synthetic or natural origin, inumerous agents can fulfill this role with a particular focus on the latter due to its notable biocompatibility and sustainable sourcing ^4^ ^,^ ^5^.

In this context, certain plant-derived agents exhibit strong interactions with biological tissues, enhancing their physicochemical properties. Among these agents, proanthocyanidins (PAC), extracted from grape seeds (Vitis vinifera), feature prominently in the literature ^5^ ^,^ ^6^ ^,^ ^7^ ^,^ ^8^ ^,^ ^9^. Despite extensive study, the precise cross-linking mechanism between collagen and PAC remains incompletely elucidated ^10^. However, four different theories have been proposed to elucidate this polyphenol chemical mechanism, suggesting interaction between PAC and collagen via covalent bonds ^8^, hydrogen bonds ^11^, ionic interactions, and hydrophobic interactions ^12^. The lack of comprehensive understanding regarding this interaction results in variability in application techniques, formulations and concentrations of this biomodifying agent.

Therefore, understanding the solubility of polyphenols is crucial, as it holds relevance across various domains such as materials science, pharmaceuticals, and environmental studies. Notably, when crafting pharmaceuticals, attention to aqueous solubility is indispensable, given its significant impact on pharmacokinetic properties like absorption, distribution, metabolism, and excretion. Several factors can influence the solubility and chemical interaction of organic and inorganic compounds (concentration, pressure, temperature and pH) ^13^. In this study, three distinct pH levels were employed to assess crosslinking efficacy, with acidic ^12^ and neutral ^13^ ^,^ ^14^ solutions previously noted for their effectiveness. However, the use of alkaline solutions for this purpose remains unexplored. Given the limited prior research, evaluating the solvent pH's influence on the action of a natural agent is imperative for refining techniques. Thus, this study aims to ascertain the effectiveness and stability of the biomodifying effect of 6.5% grape seed extract (GSE) solutions across different pH levels when applied to dentin collagen. Our study hypothesizes that (i) there will be no variance in the elasticity modulus and bond stability among groups treated with grape seed extract at different pH levels and (ii) no disparity will exist in the chemical absorbance bands among the tested groups.

## Materials and Methods

### Study design

**Figure 1:**
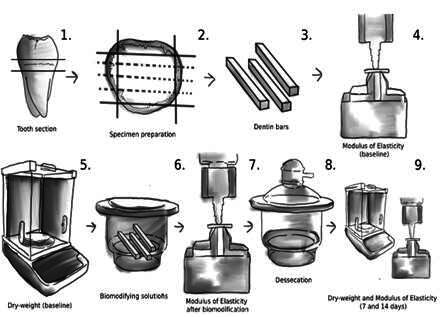
Flowchart of the study design.

This study was approved by the Research Ethics Committee of the Federal University of Ceará (# 3,212,734). It is an *in vitro* laboratory study using 6.5% grape seed extract, which was solubilized in deionized water and stabilized at different pHs: acid (pH=4.42), neutral (pH=6.96) and alkaline (pH=11.92), with distilled water (pH=6.75) as control. The dependent variables were modulus of elasticity and mass variation, quantitatively evaluated by three-point flexural test and analytical scale, respectively (Figure 1). A total of ten specimens were prepared for each experimental group. Qualitative analyses were performed with Infrared and Raman spectroscopies.

### Specimen Preparation

Forty caries-free human third molars were collected after obtaining the patients’ informed consent. The teeth were cleaned and stored in 0.1% thymol solution for a month. Using a water-cooled low-speed diamond disc (Extec Division Excel Technologies Inc.) mounted in a sectioning machine (Isomet 1000®, Buehler, Lake Bluff, United States), the teeth were cut perpendicular to the long axis of the root at two sites to obtain a 0.5 mm thick dentin disc. Subsequently, cuts were made in order to create dentin beams measuring approximately 1.7 mm x 6.0 mm x 0.5 mm. Then, the specimens were immersed in a 10% phosphoric acid solution for five hours, under constant stirring at room temperature ^6^.

### Solution Preparation

Grape seed extract (GSE, Vitis vinifera, Mega-Natural Gold; 95% Polyphenols, California, United States) was used to prepare the solutions. Initially, GSE was weighed to prepare 20 mL at a 6.5% (w/v) concentration ^10^. Next, dissolution was performed in deionized water, and the pH was measured, obtaining a solution with pH= 4.42 (acidic). To prepare the alkaline solution, the same amount of GSE was dissolved in deionized water; then, 1M NaOH was added until an alkaline solution was obtained (pH=11.92). Subsequently, water was added up to the previously determined amount (20 mL). For neutral solutions, GSE was dissolved in deionized water, then phosphate buffer saline (NaCl 137 mM, Phosphate 10 mM, KCl 2.7 mM, pH 7.4) was used to stabilize the pH close to 7.0 (pH=6.96). The solutions were kept under stirring at room temperature for 30 min until complete dissolution of the polyphenols.

### Modulus of Elasticity (ME)

According to the protocol by Aguiar et al. (2014), the demineralized dentin bars were submitted to the three-point flexural test using a universal testing machine (Instron 3345, Canton, São José dos Pinhais, Brazil), with a speed of 0.5 mm/min and a 5 N load cell, thus obtaining the baseline values. The specimens were immersed for 1 hour in the respective biomodifying solutions and subsequently rinsed with distilled water for 30 seconds, and a new measurement of the modulus of elasticity was performed. After that, the specimens were stored in artificial saliva (1.5 mM CaCl2, 0.9 mM NaH2PO4, 0.13 M KCl and 5 mM NaN3 buffered at pH 7.0 with HEPES buffer) at 37°C to simulate an intraoral setting. After 7 and 14 days storaging, new measurements of the modulus of elasticity were performed. The storage solution was changed daily, and its pH was monitored at every change.

### Mass variation

The same specimens used to measure the modulus of elasticity were dry weighed before and after their biomodification with an analytical scale with a precision of five decimal places (0.01mg accuracy, AUX-220, Shimadzu®, Japan). Before each measurement, the bars were dehydrated in a vacuum desiccator containing colloidal silica for 24 hours at room temperature and evaluated after 7 and 14 days ^6^.

### Fourier transform infrared spectroscopy (FT-IR)

Specimen with similar dimensions to those described in the previous assays were prepared for qualitative analysis (n=3) in Fourier transformed infrared spectroscopy (Attenuated Total Reflectance - Durascope, Smiths Detection; Nicolet‐380, Nicolet, United States). All dentin bars were subjected to its determined biomodification groups for 1 hour. Before absorbance reading, the specimens were vacuum dried for 24 hours. The measurement consisted of the acquisition of an absorbance spectrum in the region between 4000-400cm^-1^ with a resolution of 0.5 cm^-1^ and 32 scans for each acquired spectra. Readings were performed in the central region of the specimens, ensuring complete coverage of the crystal.

### Raman Spectroscopy (FT-Raman)

The same specimens submitted to FT-IR were subjected to qualitative analysis in FT-Raman (Vertex 70-RAM II, Bruker Analytical, Karlsruhe, Germany), thus acting as a complementary analysis. For this purpose, a laser with a wavelength of 1064 nm with an acquisition time of 120 minutes and a spectrum of 0-4000 cm^-1^ was used. The area selected for reading was located in the center of the specimen. All readings were performed in an environment protected from light penetration.

### Statistical Analysis

For statistical analysis of the quantitative data, a Shapiro-Wilk normality test was performed. To establish an intergroup comparison, a two-way ANOVA test for repeated measures was used. Differences between groups were analyzed using Tukey's *post hoc* test (SigmaPlot 14.0, SYSTAT, Chicago, United States). All tests were performed with a significance level of 5% (p<0.05).

## Results

Following biomodification, all tested groups exhibited an increase in the modulus of elasticity (ME). At seven-day period, tested groups experienced a decline in the ME.values (p<0.001). However, the group treated with the acidic solution did not maintain stable ME levels post-biomodification, experiencing consistent decreases after 7 and 14 days of storage. Conversely, GSE dissolved in an alkaline and neutral solvent demonstrated stability between 7 and 14 days (p=0.984 and p=0.198, respectively). Inter-group analysis revealed a significant difference between the acidic solution group and the others post-biomodification (p<0.001). However, after 14 days, no significant difference was observed between the acidic solution group and the group immersed in an alkaline solution (p=0.971). Additionally, there was no significant difference between the neutral solution group and the control group after seven days of storage (p=0.743) (Table 1).

**Table 1:** Mean (SD) of modulus of elasticity values, in MPa, of dentin bars submitted to immersion in grape seed extract solution stabilized at different pHs and storing time (before and after biomodification).

	Baseline	Biomodification	7 days	14 days
Control	1.41 (0.34)^Aa^	1.53 (0.50)^Da^	1.70 (0.47)^Ca^	0.96(0.43)^Ba^
Acid	1.01 (0.35)^Ad^	19.37 (3.12)^Aa^	12.21 (3.81)^Ab^	5.03(0.78)^Ac^
Neutral	1.22 (0.47)^Ab^	6.84 (0.94)^Ca^	2.52 (0.68)^BCb^	1.46 (0.52)^Bb^
Alkaline	1.11 (0.24)^Ac^	10.45 (1.59)^Ba^	4.37 (0.96)^Bb^	4.67 (0.90)^Ab^

Different capital letters indicate statistical differences in column (p<0.05). Different lowercase letters indicate statistical differences in rows (p<0.05).

Regarding mass variation, inter-group analysis revealed that the group treated with an acidic solution exhibited a greater weight variation than the control group (p<0.001) post-biomodification. Similarly, this difference maintained after 14 days (p=0.005). However, in the intra-group analysis comparing baseline to 14 days, no significant differences were observed among all groups (p>0.05) (Table 2).

**Table 2:** Mean (SD) of mass values, 10^-4^g, of dentin bars submitted to immersion in grape seed extract solution stabilized at different pHs and storing time (before and after biomodification).

	Baseline	Biomodification	7 days	14 days
Control	38.41 (5.36)^Aa^	34.92 (5.77)^Ba^	33.96 (5.33)^Aa^	33.35 (5.03)^Ba^
Acid	37.31 (3.70)^Ab^	43.77 (4.41)^Aa^	39.15 (3.96)^Aab^	40.16 (3.81)^Aab^
Neutral	37.01 (5.21)^Aa^	38.81 (5.50)^ABa^	35.14 (4.65)^Aa^	34.96 (5.11)^ABa^
Alkaline	36.61 (3,63)^Aa^	38.86 (3.52)^ABa^	36.31 (3.04)^Aa^	35.45 (4.00)^ABa^

Different capital letters indicate statistical differences in column (p<0.05). Different lowercase letters indicate statistical differences in rows (p<0.05).

**Figure 2:**
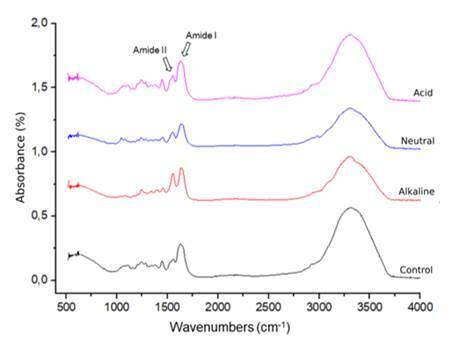
Fourier Transform Infrared Spectroscopy of the collagen bars after treatment with the respective tested solutions. Amide I and II peaks highlighted (ARROWS).

**Figure 3:**
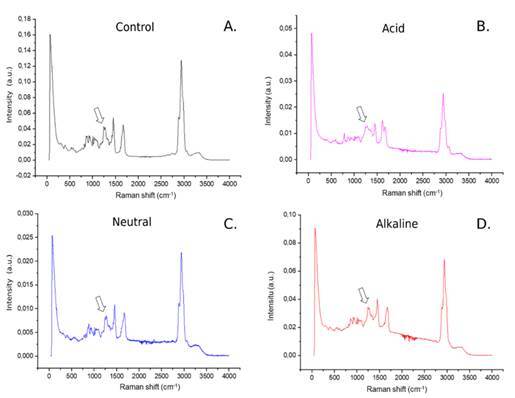
FT-RAMAN absorption spectrum from 0 - 4000cm^−1^ (A). Collagen and amide III absorption spectrum of 1200 - 1280cm^−1^ (ARROW) specimens (B). CO3 and amide II absorption spectrum of 1500 - 1600cm^−1^ specimens (C). C-H radical and water absorption spectrum of 2500 - 3500cm^‐1^ specimens (D).

Fourier transform infrared spectroscopy (FT-IR) analysis showed that the alkaline and acidic solutions led to an increase in the peak corresponding to amide II (~1560 cm^-1). Additionally, in the alkaline and neutral groups, a decrease in the peak within the range of 3200-3600 cm^-1 indicated a reduction in the number of free hydroxyl groups (O-H). The lower 500-1000 cm^-1 (PO4^-3) band absorbance present in all groups suggested complete demineralization of the tested specimens (Figure 2). In the analysis of Raman spectroscopy (FT-Raman), the peak associated with amide III (~1240 cm^-1) was more pronounced in all experimental groups, a feature not observed in the FT-IR analysis. Notably, the group treated with the control and acidic solution exhibited a lower absorbance and displacement of this peak towards regions closer to 1500 cm^-1 (Figure 3).

## Discussion

Fundamental chemical properties of a solvent, such as pH, can significantly impact the effectiveness of cross-linking reagents ^14^. However, to our knowledge, prior studies have not explored how pH affects the interaction between natural reagents and dentin collagen. Determining the optimal pH is crucial for determining a reliable and efficient technique. In this study, the authors selected proanthocyanidins (PAC) at 6,5% as a biomodifying agent due to its demonstrated high solubility in water across the tested pH ranges and its well documented biomodifying properties ^13^ ^,^ ^14^.

While several other polyphenols, such as ellagic acid, hesperidin, and apigenin, are soluble in alkaline solutions, they exhibit limited solubility in neutral solvents like water. It's worth noting that grape seed extract contains a diverse array of polyphenols, forming agglomerates and complex aggregates with 64 different structures. These agglomerates possess varied properties depending on the solvent used, and many of them are insoluble in organic solvents. Polymeric PACs, for instance, encounter significant solubilization challenges in polar organic solvents ^15^. Therefore, investigating the influence of pH on the interaction between collagen and biomodifying agents is necessary to expand the range of polyphenols that can be utilized to preserve resin-dentin bonds.

The initial null hypothesis was rejected because, following biomodification, the group treated with the acidic solution displayed superior modulus of elasticity values compared to the others. This outcome is consistent with the findings of Liu et al. ^12^, who demonstrated that PAC enhances biological stability by reinforcing the collagen network in an acidic environment. Interestingly, the ME control group results in this study remained unaltered throughout the testing period, a phenomenon not observed in the other groups. It is likely that the decrease in elastic modulus post-storage was not due to collagen degradation, but rather to the hydrolysis of bonds formed during crosslinking. Many studies employing similar methodologies utilize collagenases to induce collagen fibril degradation ^4^ ^,^ ^15^. However, these enzymes do not allow us to determine whether the decline in modulus of elasticity arises from the loss of newly formed crosslinks or the degeneration of preexisting bonds within the collagen fibril.

After seven days of storage, all tested groups presented a decrease in ME. It can be explained by the presence of galloylated and non-galloylated groups in GSE. The galloylated groups promote an intense initial bond but undergo hydrolysis in the ester bonds. On the other hand, non-galloylated molecules remain stable for longer periods but present less reactivity ^13^ ^,^ ^16^. After 14 days of dentin collagen immersion in artificial saliva, the groups treated with acidic and alkaline solutions were effective in maintaining the high modulus compared to the baseline. Since a ME increase after biomodification is probably due to collagen crosslinking, it is important to note that crosslinks can be degraded, but the collagen itself remains stable. Therefore, the authors infer that both solutions could be useful for immediate collagen crosslinking. However, the group treated with an acidic solution did not remain stable during the tested periods. Probably this occurred due to the acid medium being capable of promoting a breakdown of type B interflavine bonds found in PAC ^16^ ^,^ ^17^. These bonds are less stable when exposed to low pH solutions, thus impairing the activity of this polyphenol ^13^ ^,^ ^18^.

Although the present study employed low concentrations of NaOH, it's important to acknowledge that a solution with a high pH can have detrimental effects on dental pulp. Therefore, caution must be exercised to prevent direct contact of the solution with connective tissue. Consequently, partial neutralization of the solution with weak acids is advisable prior to any potential clinical application ^19^. It's worth noting that PACs are biocompatible and have the capability to promote mineralization at both the intrafibrillar and interfibrillar levels through cell stimulation ^20^ ^,^ ^21^ ^,^ ^22^.

Regarding mass variation, there was no statistically significant difference observed in all groups, except for the acidic group, across different measurement times, probably due to the higher H+ release of acidic solutions may reduce the mineral content for the dentin collagen overtime ^2^ ^,^ ^4^. This can be attributed to the large dimensions of the specimens and the slow rate of collagen degradation, resulting in minimal mass variation. Additionally, the thickness of the bars (0.5mm) contributed to the prolonged application time of the tested substance (1 hour), which may not align with clinically reliable protocols, highlighting a limitation of the present study. As a secondary observation, it's noteworthy that the significant pigmentation induced by the use of grape seed extract was more pronounced in the groups treated with acidic and alkaline solutions, a finding also documented by Moreira et al. ^22^.

The FT-IR and FT-Raman spectra are indicative of the conformational changes in collagen molecules. Hence, these methods are routinely employed to assess alterations in collagen structure following crosslinking. The absorption peaks of collagen, namely amide I, amide II, and amide III, are key indicators. The amide I peak at ~1650 cm^-1 primarily corresponds to stretching vibrations of the C = O groups, while the amide II peak at ~1560 cm^-1 is largely attributed to N-H bending vibrations coupled with C-N stretching and CH2 bending vibrations. Additionally, the amide III peak at ~1240 cm^-1 predominantly arises from C-N elongation, N-H bending vibrations, and vibrational modes of CH2 groups on the glycine main chain and proline side chains. These three peaks serve as reliable indicators of crosslinking in collagen. Post-crosslinking, the absorption peak of amide II intensifies, while that of amide I remains unchanged ^3^ ^,^ ^23^. Consequently, the ratio of amide I to amide II signifies the extent of bond formation during the crosslinking process. Notably, in the group treated with an alkaline solution, the ratio between the peaks undergoes significant alteration, indicating that this solution facilitates a greater potential for crosslinking.

Regarding FT-IR and FT-Raman results, similar outcomes to those found by Liu et al. ^12^ were observed. It demonstrated an increase of amide II peak in both acid and alkaline groups. This fact can be attributed to the C-C aromatic ring stretching in the PAC at ~ 1517 cm^-1^, indicating an interaction between the terminal bonds of the amino acids and the tested polyphenol. In FT-IR, the neutral and alkaline solutions presented a decrease in amide III peaks (~ 1240 cm^-1^). No literature has attributed the peak at ~1240 cm^-1^ to specific vibration modes but evidence has indicated that this may be due to the CH_2_ bending of glycine 33 residues, as well as symmetrical elongation of the carboxylate (COO-) side chains from glutamate and aspartate residues. The reduction of the peak between 3200-3600 in the neutral and alkaline groups indicates a decrease in free O-H bonds since it presented a reduction in the amount of water in the sample. This is a desired effect since the approximation between the molecules of the triple-helix chain prevents the accumulation of water in this region by a steric impediment. Therefore, a hydrolytic stability of the bonds could be also expected ^12^. Additionally, this finding may indicate a saturation of the hydrogen-PAC bond. In FT-Raman, there was a change in the peak referring to amide III in the group immersed in all solution groups, a fact not observed in FT-IR, indicating that the association of both methodologies is essential for a correct analysis of the bonds in collagen ^23^. In view of the variation of peaks, it can be inferred that the second hypothesis was also rejected.

Indirectly, the application of GSE may inhibit the action of collagenolytic enzymes, such as matrix metalloproteinases ^24^, which are activated in an acidic environment ^22^ ^,^ ^24^. However, the present study did not evaluate the enzymatic activity in collagen. Probably, the use of alkaline solutions can reduce the degradation by this process, since there would not have a proper pH reduction for protease activation. With that said, further studies must be carried out to confirm this activity. Another relevant issue is the heterogeneity of dentin under clinical conditions, which entails variables not analyzed in the study, such as the buffering effect of dentin fluid on the solution and the interference in the polymerization of adhesive monomers, especially when high concentrations of PAC are used ^25^. Therefore, randomized clinical trials are crucial to verify the effectiveness of this technique.

## Conclusion

It is concluded that the pH of the solution directly influences the action of the grape seed extract, with promising results found in both acidic and alkaline solutions, as they improve the mechanical property of collagen, whereas the alkaline GSE solution remains stable after 14 days of storage.
